# Plants change their clocks to flower at the right time

**DOI:** 10.1073/pnas.2208745119

**Published:** 2022-07-11

**Authors:** Stephen Pearce

**Affiliations:** ^a^Sustainable Soils and Crops, Rothamsted Research, Harpenden, Hertfordshire AL5 2JQ, United Kingdom

Humanity has a major stake in the reproductive success of plants, especially of crops such as rice and wheat, which are important sources of calories and protein in our diets. Because annual plants have just one opportunity to produce seeds, it is essential that this is timed to coincide with optimal environmental conditions. To maximize their chances of success, plants have evolved a series of intricate mechanisms to monitor changes in their environment and to adjust their development accordingly (1). In a study published in PNAS, Andrade et al. (2) describe important advances in our understanding of how plants regulate their reproduction in response to one critical environmental signal: photoperiod.

## Flowering Plants and Photoperiodism

Whereas temperature can fluctuate wildly, the time between dawn and dusk changes consistently and predictably at a given latitude, making photoperiod a reliable seasonal cue by which plants can regulate their development. To measure photoperiod, plants absorb photons of sunlight in their leaves, activating phytochromes, a class of photoreceptor. The circadian clock measures and integrates these light signals to ensure that the synthesis of florigenic proteins occurs only under appropriate photoperiods. The translocation of florigens from the leaves to the shoot apical meristem triggers flowering and marks the irreversible transition to reproductive development (1).

The core components of this photoperiod response system are conserved across flowering plants, even between species that are adapted to a wide range of different latitudes. For example, flowering plants can thrive in equatorial regions where they experience near-equal day lengths year-round as well as in places such as Greenland and Tierra del Fuego that are characterized by major seasonal variation in photoperiod.

Adapting to such variable environments requires that each plant species tailors this common system to regulate the photoperiod-dependent synthesis of florigens in a way that is optimal for reproduction in local conditions. “Short-day” plants, such as rice and soybean, are induced to flower in winter months, ensuring that they are well adapted to the tropical and subtropical regions where they are primarily grown. By contrast, “long-day” plants, such as the temperate cereals barley and wheat, initiate flowering during spring in response to lengthening photoperiods. Although this historical classification of plants according to the day length in which they flower is still commonly used (3), we now know that plants respond to photoperiod by measuring the length of the night (4).

The study by Andrade et al. (2) describes the characterization of circadian clock components in rice, demonstrating their importance in integrating photoperiod signals to regulate flowering time. These findings suggest a model whereby functional differences in the genes targeted by the circadian clock might contribute to plant adaptation to different environments. This work provides a framework to develop testable hypotheses to characterize variation in photoperiod response in other plants that can be applied to help breed crop varieties better adapted to current and future environments.

## The Evening Complex in Rice

In their study, Andrade et al. (2) demonstrate that the *EARLY FLOWERING 3* (*ELF3*) and *LUX ARRHYTHMO* (*LUX*) genes, which encode components of the circadian clock known as the Evening Complex, are critical regulators of photoperiodism in rice. When wild-type plants are grown in short-day photoperiods, transcript levels of several floral repressors are down-regulated, resulting in higher expression of genes encoding florigenic proteins and accelerated flowering. By contrast, when mutant plants lacking any functional copies of either *ELF3* or *LUX* are grown in the same conditions, transcript levels of floral repressors remain high, and genes encoding florigenic proteins are expressed at low levels. None of these mutant plants flower, regardless of photoperiod, showing that the Evening Complex is essential for flowering in rice. These results are consistent with an earlier study demonstrating the importance of the Evening Complex for flowering in soybean (5).

Both ELF3 and LUX binding sites are enriched in the cis-regulatory regions of floral repressor genes, suggesting that the Evening Complex regulates flowering by directly suppressing these genes. To explore how photoperiod signals are integrated into this system, the authors measured the accumulation of Evening Complex proteins in different conditions. In a process requiring PHYTOCHROME B, exposure to light triggered the posttranslational modification of ELF3 proteins to a proposed nonfunctional form that is unable to suppress the expression of its target genes.

**Fig. 1.**
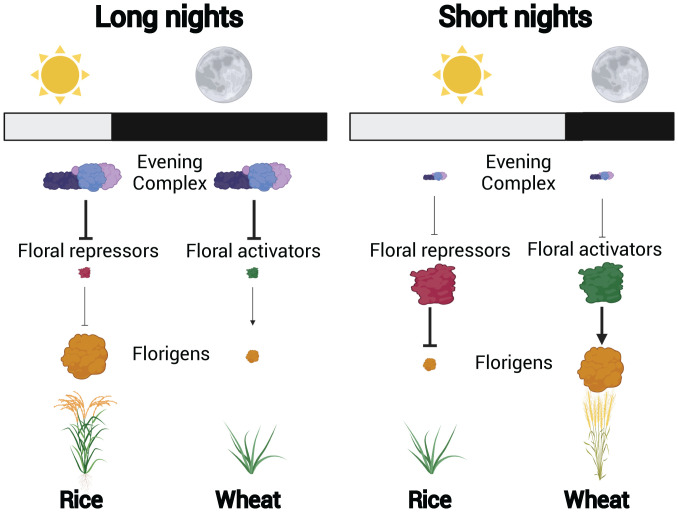
A simplified model for the hypothesized role of the Evening Complex in photoperiodism. In long nights, functional Evening Complex proteins accumulate, increasing the suppression of their target genes. For short-day plants, such as rice, the Evening Complex suppresses floral repressors, resulting in increased florigen synthesis and accelerated flowering. For long-day plants, such as wheat, the Evening Complex suppresses floral activators, resulting in reduced florigen synthesis and delayed flowering. In short nights, the levels of functional Evening Complex proteins are much lower, resulting in the inverse response.

According to this model, functional ELF3 proteins accumulate during long nights, forming complexes with LUX that directly suppress the transcription of floral repressors, resulting in the synthesis of florigen that induces flowering (Fig. 1). When night length falls below a critical threshold, the levels of functional ELF3 proteins are insufficient to suppress floral repressors, delaying flowering. Thus, the Evening Complex measures night length to regulate the photoperiod-dependent expression of floral repressor genes, ensuring that rice plants flower in short-day photoperiods that are optimal for their reproductive success.

This model is consistent with observations from “night-break” experiments, whereby rice plants that are exposed to a 15-min period of light during a long night do not flower (6). The rapid activation of phytochromes in response to night break is likely sufficient to induce posttranslational modification of ELF3, delaying flowering. In future studies, it will be important to decipher the nature of the posttranslational modification of ELF3 and to study the dynamics of the conversion between ELF3 protein forms in response to the gradual changes in photoperiod experienced by plants in farmers’ fields.

## The Evening Complex in Long-Day Plants

Can these findings in rice help explain the opposite photoperiod responses of long-day plants? Whereas loss-of-function mutations in Evening Complex genes abolish flowering in rice, similar alleles accelerate flowering in wheat and barley (7, 8). As the authors postulate, this divergent response might be explained by differences in the function of genes targeted by the Evening Complex that arose as these plants underwent adaptation to different environments. For short-day plants, the accumulation of functional Evening Complex proteins during long nights signals the requirement to accelerate flowering, so this complex suppresses floral repressors. Under the same conditions, plants adapted to long days require the opposite response, so have evolved an Evening Complex that instead suppresses genes that function as floral activators in these species (Fig. 1). This hypothesis can be tested directly in different long-day plants. This model is consistent with characterized Evening Complex targets in *Arabidopsis* (9) and in wheat, where variation in *ELF3* affects the transcript levels of the floral activator *PHOTOPERIOD 1*, which may be a direct target of the Evening Complex (7, 10).

## Adapting Crops to Current and Future Environments

The knowledge derived from a more detailed understanding of photoperiod-regulated gene networks can be applied to breeding and germplasm development. Flowering time and photoperiod sensitivity are among the most important factors for adapting crop varieties to different environments and are major determinants of yield (11). During decades of formal and informal breeding, the selection for genetic variation in photoperiod-response pathways has contributed to a remarkable expansion of the range of environments in which crops can be grown. Common wheat was domesticated in the Fertile Crescent but is now cultivated from 65°N to 45°S (12). Likewise, soybean varieties belonging to different maturity groups are today well adapted to latitudes ranging from 50°N to 35°S (13). Characterizing the Evening Complex and its downstream regulatory pathways in different crops will help researchers better understand the basis of crop adaptation and allow breeders to more easily select for specific genetic variants associated with photoperiod responsiveness during variety development. The exploitation of natural genetic variation in germplasm collections (14) and the use of precision editing tools to increase genetic diversity in specific pathways (15) represent powerful and complementary approaches to fine-tune photoperiod-responsive gene networks. These approaches can help develop genotypes that are optimally adapted to a target environment and might further expand the range of crop cultivation, especially for understudied crops that have only a short history of formal breeding.

Furthermore, flowering time is also impacted by ambient temperature. Although photoperiod does not change year to year, mean temperatures in most agricultural regions will likely continue to rise for the next several decades, contributing to the steady acceleration of flowering time observed since the dawn of the industrial age (16, 17). It will be interesting to determine whether and to what extent ambient temperature signals are integrated by the Evening Complex to regulate flowering time in crop plants (18, 19). Characterizing these pathways will help breeders to identify germplasm that is more resilient to changing temperatures and to facilitate the selection of varieties that can sustain high yields in future environments.
